# BMP-2 Up-Regulates PTEN Expression and Induces Apoptosis of Pulmonary Artery Smooth Muscle Cells under Hypoxia

**DOI:** 10.1371/journal.pone.0035283

**Published:** 2012-05-15

**Authors:** Weifeng Pi, Xuejun Guo, Liping Su, Weiguo Xu

**Affiliations:** 1 Department of Respiratory Medicine, Xinhua Hospital, School of Medicine, Jiaotong University, Shanghai, China; 2 Department of Bioengineering, National University of Singapore, Singapore, Singapore; Leiden University Medical Center, The Netherlands

## Abstract

**Aim:**

To investigate the role of bone morphogenetic protein 2 (BMP-2) in regulation of phosphatase and tensin homologue deleted on chromosome ten (PTEN) and apoptosis of pulmonary artery smooth muscle cells (PASMCs) under hypoxia.

**Methods:**

Normal human PASMCs were cultured in growth medium (GM) and treated with BMP-2 from 5–80 ng/ml under hypoxia (5% CO_2_+94% N_2_+1% O_2_) for 72 hours. Gene expression of PTEN, AKT-1 and AKT-2 were determined by quantitative RT-PCR (QRT-PCR). Protein expression levels of PTEN, AKT and phosph-AKT (pAKT) were determined. Apoptosis of PASMCs were determined by measuring activities of caspases-3, -8 and -9. siRNA-smad-4, bpV(HOpic) (PTEN inhibitor) and GW9662 (PPARγ antagonist) were used to determine the signalling pathways.

**Results:**

Proliferation of PASMCs showed dose dependence of BMP-2, the lowest proliferation rate was achieved at 60 ng/ml concentration under hypoxia (82.2±2.8%). BMP-2 increased PTEN gene expression level, while AKT-1 and AKT-2 did not change. Consistently, the PTEN protein expression also showed dose dependence of BMP-2. AKT activity significantly reduced in BMP-2 treated PASMCs. Increased activities of caspase-3, -8 and -9 of PASMCs were found after cultured with BMP-2. PTEN expression remained unchanged when Smad-4 expression was inhibited by siRNA-Smad-4. bpV(HOpic) and GW9662 (PPARγ inhibitor) inhibited PTEN protein expression and recovered PASMCs proliferation rate.

**Conclusion:**

BMP-2 increased PTEN expression under hypoxia in a dose dependent pattern. BMP-2 reduced AKT activity and increased caspase activity of PASMCs under hypoxia. The increased PTEN expression may be mediated through PPARγ signalling pathway, instead of BMP/Smad signalling pathway.

## Introduction

Hypoxic pulmonary hypertension, one of the most common pulmonary arterial hypertension, is characterized by increased proliferation and reduced apoptosis of smooth muscle cells [1]. It contributes to increased pulmonary vascular resistance and increased pulmonary artery pressure. It leads to severe chronic obstructive pulmonary disease and right ventricular failure [2]. Since the proliferation of pulmonary artery smooth muscle cells (PASMCs) is an essential feature of vascular proliferative disorders, considerable effort has been made to develop therapeutic strategies to effectively suppress smooth muscle cells (SMCs) proliferation.

Bone morphogenetic protein 2 (BMP-2), which belongs to the transforming growth factor beta super-family, is a negative regulator of PASMC growth [3]. It is a potent inhibitor of vascular SMCs proliferation both in vitro and in vivo [3]. BMP-2 has a potent inhibitory action on the growth of cultured rat aortic SMCs. In addition, transfer BMP-2 gene into an injured artery inhibited SMC proliferation in vivo as well as in vitro, using a rat carotid balloon injury model. Wong et al., [4] demonstrated that BMP-2 could inhibit PDGF-stimulated proliferation of arterial SMCs through induction of p21. Hansmann et al., [5] shows that anti-proliferative effects of BMP-2/BMP-RII signaling in primary PASMCs can be attributed to activation of PPARγ and its putative transcription target apoE.

It has been shown that PPARγ agonist up-regulated phosphatase and tensin homologue deleted on chromosome ten (PTEN) expression in allergen-induced asthmatic lungs [6]. Zhang et al., [7] demonstrated that PPAR activator rosiglitazone inhibited human hepatocarcinoma BEL-7404 cell migration via up-regulating PTEN. The tumour suppressor gene PTEN encodes a dual-specificity phosphatase that recognizes protein and phosphatidylinositiol substrates and modulates cellular functions such as migration and proliferation.

Though BMP-2 activates PPARγ to achieve anti-proliferative effects on SMCs, its role in regulation of PTEN expression in SMC proliferation is yet established. Here we report that BMP-2 increased PTEN expression of PASMCs under hypoxia in a dose dependent pattern. BMP-2 reduced AKT activity and increased caspase activity of PASMCs under hypoxia. The increased PTEN expression may be mediated through PPARγ signalling pathway, instead of BMP/Smad signalling pathway.

## Materials and Methods

### Cell Culture of PASMC

Human primary PASMC was purchased from ScienCell Research Laboratories (CA, USA). PASMC was cultured and expanded in SMC growth medium (GM): SMC basal medium (BM) (ScienCell Research Laboratories, CA USA) supplemented with SMC growth supplement (ScienCell Research Laboratories), 10% fetal bovine serum (FBS) and 1% Penicillin/Streptomycin. PASMCs were regularly passaged every 4–5 days. BMP-2 (Miltenyi Biotec, Singapore) was supplemented in the cell culture medium to determine its effect on PASMC proliferation.

bpV(HOpic) (Merck, Germany), a PTEN inhibitor, and GW9662 (Sigma Aldrich, USA), an antagonist of PPARγ, were used to determine the possible signalling pathways mediated by BMP-2.

### Hypoxia Treatment

PASMC were cultured in hypoxic condition to determine the anti-proliferative effect of BMP-2 on PASMCs. Hypoxia was created in an incubator: 5% CO_2_+94% N_2_+1% O_2_, 37°C.

PASMCs cultured in GM supplemented with BMP-2 were cultured in hypoxia incubator for 72 hours. The supernatant and cells were collected for cytotoxic, gene and protein expression studies.

### Quantitative RT-PCR (QRT-PCR) Analysis

PASMCs were analyzed by QRT-PCR to determine gene expression after treated with BMP-2. Total RNA was isolated using RNA Isolation Kit (QIAGEN, USA) according to the manufacturer’s instructions [8]. DNase I (Fermentas, USA) was used to remove DNA from total RNA. cDNA was synthesized using Maxima® First Strand cDNA Synthesis Kit (Fermentas, USA). To quantify gene expression, Maxima® SYBR Green qPCR Master Mix (2X) (Fermentas, USA) was used. The QPCR thermal cycling protocol for 40 cycles was programmed as following: 1 cycle of initial denaturation for 10 min, then denaturation at 95°C for 15 seconds, annealing for 30 seconds and extension at 72°C for 30 seconds. The primers used in the study were listed in Table 1.

**Table 1:** RT-PCR primers and annealing temperature.

GAPDH60°C, 90 bp,	forward	5' AGCCACATCGCTCAGACAC 3'
	reverse	5' TAAAAGCAGCCCTGGTGAC 3'
PTEN60°C, 155 bp	forward	5' TCACCAACTGAAGTGGCTAAAGA 3'
	reverse	5' CTCCATTCCCCTAACCCGA 3'
AKT-158°C, 125 bp	forward	5' TAACCTTTCCGCTGTCGC 3'
	reverse	5' ATGTTGTAAAAAAACGCCG 3'
AKT-258°C, 127 bp	forward	5' GGTCGCCAACAGCCTCAA 3'
	reverse	5' CACTTTAGCCCGTGCCTTG 3'
Smad-460°C, 128 bp	forward	5' CTTCAGGGGCTTCTAAAACAG 3'
	reverse	5' TATCAGAGAGGGAAGAGACCAG 3'

### Cell Proliferation

Cell proliferation rate of PASMCs was determined by CyQUANT® Cell Proliferation Assay Kit (Invitrogen, USA). Briefly, 1×10^4^ PASMC/well were seeded into 24-well plate and cultured with BM for 24 hours. The cell culture medium was changed to GM supplemented with BMP-2 for 72 hours in incubator under hypoxia. After that, cell supernatant was removed. Cells were washed with PBS and frozen at −80°C freezer for at least 1 hour. Then each well was incubated with 200 µl CyQUANT® cell-lysis buffer containing DNase-free RNase (1.35 U/ml) to eliminate the RNA component of the fluorescent signal for 1 hour at room temperature. After that, 200 µl cell lysis buffer containing 2X solution of CyQUANT® GR dye was added into each sample for 10 min. The fluorescence intensity was measured using a Tecan fluorescence microplate reader (Tecan Infinite M200, LabX Canada) at an excitation wavelength of 480 nm and an emission wavelength of 520 nm.

### Lactate Dehydrogenase (LDH) Release for Cyto-toxic Determination

The cyto-toxicity of BMP-2 towards PASMCs was determined using CytoTox-ONE™ Homogeneous Membrane Integrity Assay (Promega, USA). Briefly, 5×10^4^ PASMCs/well were seeded into 12-well plate in GM supplemented with BMP-2 for 72 hours in incubator at 37°C. After that, cell culture supernatant was collected and mixed with CytoTox-ONE™ Reagent for at least 10 min. After addition of 50 µl stop solution, the fluorescence signal was measured at an excitation wavelength of 560 nm and an emission wavelength of 590 nm.

### Western Blot Analysis of PASMCs

Protein expression levels from treated and non-treated PASMCs were determined by western blot analysis [9]. PASMCs were lysated with PhosphoSafe™ Extraction Reagent (Merck, Germany) and protein concentration was determined using Bradford reagent (Bio-Rad Laboratories, USA). Proteins were separated and transferred to nitrocellulose membrane. After washing with 10 mM Tris/HCl wash buffer (pH 7.6) containing 0.05% Tween-20, the membrane was blocked with blocking buffer (5% non-fat dry milk, 10 mM Tris pH 7.5, 100 mM NaCl, 0.1% Tween-20) for 1 hour at room temperature. After blocking, the membrane was incubated with 1∶200–1∶1,000 dilution of AKT, phosphorylated (Ser 473) AKT(pAKT), PTEN, Smad-4, and GAPDH (all purchased from Santa Cruz, USA) for overnight at 4°C. After that, anti-rabbit IgG conjugated with HRP (dilution: 1∶3, 000 – 1∶8,000) was used to detect the binding of antibodies. The binding of the specific antibody was visualized using the SuperSignal Chemiluminescent Substrate kit (Pierce, USA) and exposed to X-ray film (Pierce, USA). The film was scanned and the optical intensity of the band was quantified by Olympus Micro Image software. The concentration of each protein sample was expressed as percentage after normalizing to GAPDH (regarded as 100%).

### Apoptosis Assays

Proteins from treated and non-treated PASMC were used to determine the apoptosis of PASMCs by determining the activities of caspase -3, -8 and -9.

Caspase -3 and -8 activities were determined by Caspase -3 and -8 Assay Kits, Fluorimetric (Sigma Aldrich, CASP3F and CASP8F). The fluorescence intensity of caspase-3 was recorded at wavelength of 360 nm for excitation, and at wavelength of 460 nm for emission, while it was 360 nm of excitation, and 440 nm of emission for caspase-8. The activity of caspase was calculated as Fluorescence intensity (FI)/min/ml = ΔFlt/(t x v), where ΔFlt = difference in fluorescence intensity between time zero and time t minutes, t = reaction time in min, and v = volume of sample in ml.

Similarly, Caspase-9 activity was determined by Caspase 9 Assay Kit, Fluorimetric (EMD4Biosciences, QIA72). The fluorescence intensity was recorded at wavelength 400 nm of excitation, and wavelength 505 nm of emission. The same formula as the one used for calculation of caspase-3 activity was used to calculate caspase-9 activity.

### Transfection of PASMC Using Lipoplexes Carrying pEGFP or siRNA-Smad-4

Trypsinized PASMCs were seeded at a density of 1×10^5^cells/well in 12-well plates and cultured with SMC growth medium without antibiotics. A plasmid carrying enhanced green fluorescent protein (pEGFP) was used to transfect PASMCs to optimize the transfection condition [8], [10]. After optimization, pEGFP was replaced with a plasmid carrying siRNA-Smad-4 (Invitrogen, USA) to inhibit the Smad-4 gene expression, or a negative control siRNA (siRNA-control, Invitrogen, USA).

The lipoplexes were manufactured by mixing lipofectamine-2000 (Lipo) (Invitrogen, USA) with pEGFP (2 ug) from 1∶1 to 4∶1 (volume/weight: ul/ug). Lipofectamine and plasmid DNA were diluted in 50 µl Opti-MEM® I Reduced Serum Medium (Invitrogen, USA). The pEGFP lipoplexes were developed by mixing the respective solutions. After mixing, the mixture was vortexed for 10 seconds followed by centrifuge at lowest speed for 10 minutes. Then lipoplex mixture was sedated for 10 min at room temperature and added into cell culture medium (SMC growth medium without antibiotics) to transfect PASMCs for 24 hours at 37°C in incubator.

siRNA-Smad-4 or siRNA-control lipoplexes were developed by replacing pEGFP with plasmid siRNA-Smad-4 or siRNA-control. The volume of lipofectamine used would be the one that resulted in the highest EGFP gene transfection efficiency. Next, lipofectamine volume was fixed and siRNA concentration was adjusted fom 40 nM up to 200 nM to identify the optimal ratio between lipofectamine and siRNA-Smad-4 to inhibit Smad-4 gene expression.

### Statistic Analysis

All statistical analyses were performed using SPSS (version 10.0). The data were presented as mean± standard error means (SEM) and analyzed by the method of analysis of variance (ANOVA) using Bonferroni test. All tests were performed with a significance level of 5%.

## Results

### Dose Dependent Effects of BMP-2 on PASMC Proliferation

**Figure 1:**
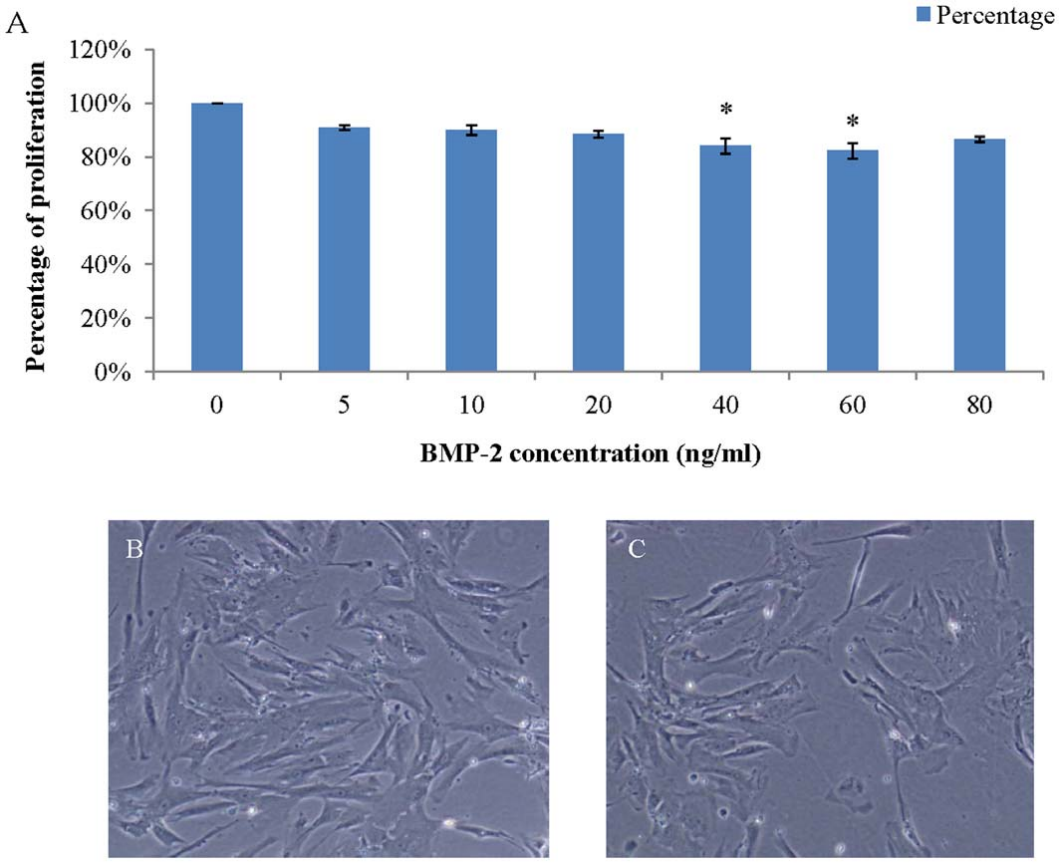
Reduced PASMCs proliferation rate as a function of BMP-2 concentration when cultured in GM under hypoxia (A). PASMCs cultured in GM supplemented with 0–80 ng/ml BMP-2. BMP-2 at the concentrations of 40 and 60 ng/ml significantly inhibited PASMC proliferation as compared with GM without BMP-2. Typical pictures of PASMCs cultured in GM only (**B**), or GM supplemented with BMP-2 at 40 ng/ml (**C**). (*: vs 0 ng/ml only, p<0.05) **(**Magnification = 100×).

The proliferative rate of PASMCs showed dose dependence of BMP-2 when PASMCs was cultured under hypoxia (Figure 1A&B). The number of PASMCs treated with 5 ng/ml BMP-2 was reduced to 90.8±1% of non-treated PASMCs (regarded as 100%), while they were 92.9±1.8% with 10 ng/ml, 88.4±1.2% with 20 ng/ml, 84.1±2.8% with 40 ng/ml, 82.2±2.8% with 60 ng/ml, 86.6±1% with 80 ng/ml. The proliferation of PASMCs was significantly reduced at 40 and 60 ng/ml concentrations of BMP-2 compared with that cultured in GM medium only.

### Toxicity of BMP-2 Towards PASMCs

**Figure 2:**
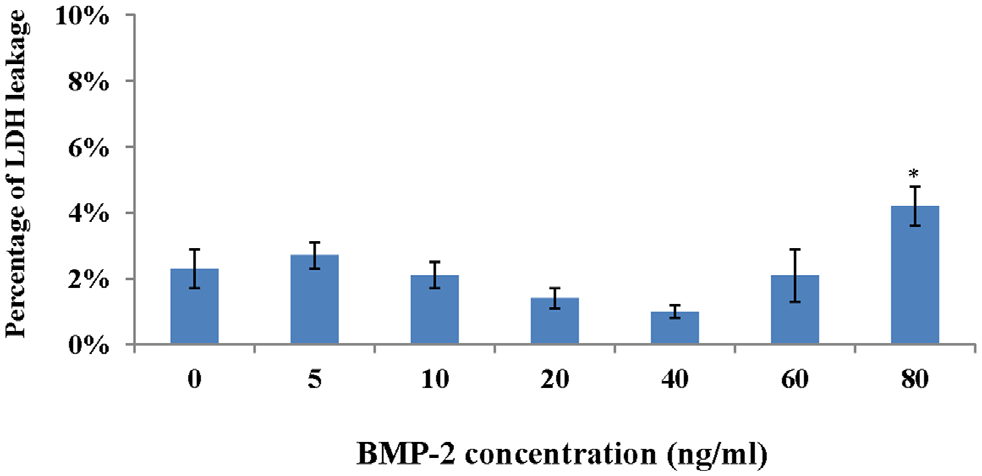
Toxicity of BMP-2 towards PASMCs when PASMCs were cultured in GM supplemented with 0−80 ng/ml BMP-2 under normoxia. It appears that only at 80 ng/ml concentration of BMP-2 resulted in significantly increased LDH leakage as compared with GM with 0 ng/ml BMP-2. The percentage of LDH leakage was normalized to fresh GM (consider as 0%). (*: vs 0 ng/ml, p<0.05).

The toxicity of BMP-2 towards PASMCs was determined by measuring LDH in the cell culture supernatant. No significant cell injury was found when BMP-2 was increased up to 60 ng/ml (Figure 2).

The percentage of LDH in supernatant was 2.3±0.6% when PASMCs were cultured in GM without BMP-2. They were 2.7±0.4% with 5 ng/ml BMP-2, 2±0.4% with 10 ng/ml, 1±0.3% with 20 ng/ml, 1±0.2% with 40 ng/ml, 2±0.8% with 60 ng/ml. However, the leakage of LDH increased to 4.2±0.6% when BMP-2 was 80 ng/ml concentration, which was significantly increased as compared with that cultured in GM medium only (Figure 2).

### BMP-2 Significantly Increased PTEN Gene Expression

**Figure 3:**
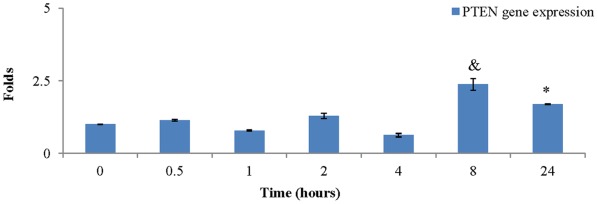
Addition of BMP-2 (40 ng/ml) in GM increased PTEN gene expression of PASMCs. BMP-2 significantly increased PTEN expression at 8 and 24 hours after treatment. (&: vs any other time point, p<0.05; *: vs 0, 1, and 4 hours, p<0.05).

BMP-2 significantly increased PTEN gene expression at 8 hours (2.4±0.2 folds, p<0.05) compared to any other time point (Figure 3). Though it reduced at 24 hours (1.7±0.02 folds), it was still significantly higher than those at baseline, 1 and 4 hours after treatment (Figure 3).

### BMP-2 did not Significantly Change AKT-1 and AKT-2 Gene Expression

**Figure 4:**
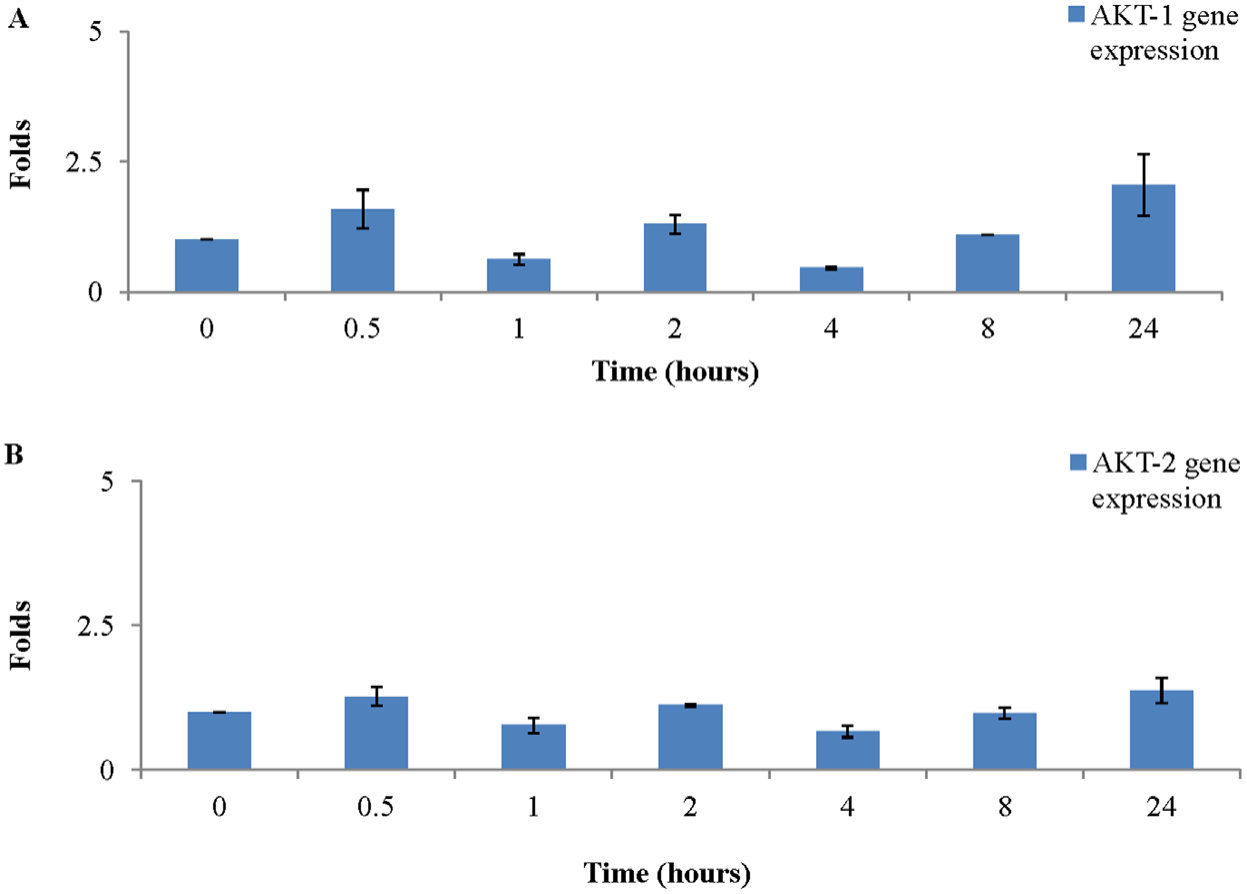
Addition of BMP-2 (40 ng/ml) in GM did not significantly change AKT-1 and AKT-2 gene expressions of PASMCs. No significant reduction or increment of AKT-1 gene expression was found when PASMCs were cultured in GM supplemented with BMP-2 (**A**). Similarly, no significant change of AKT-2 gene expression was found when PASMCs were cultured in GM supplemented with BMP-2 (**B**).

Generally, no significant reduction or increment of AKT-1 gene expression was found after addition of BMP-2 (Figure 4A). A similar pattern was also found in AKT-2 gene expression. The highest gene expression of AKT-2 was found at 24 hours (1.4±0.22 folds) as compared with baseline. However, no significant change was found after addition of BMP-2 (Figure 4B).

### Western Blot Analysis of PASMCs Cultured in GM Supplemented with BMP-2

**Figure 5:**
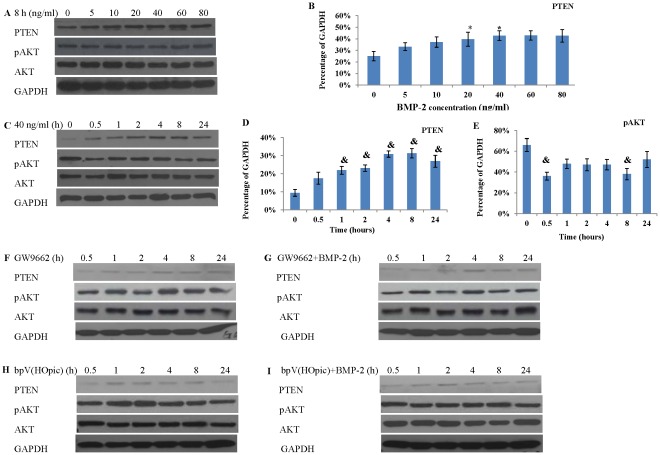
Western blot analysis of PASMCs cultured in GM supplemented with BMP-2 under hypoxia. (**A**) A dose dependent effect of BMP-2 on PTEN protein expression when BMP-2 was supplemented up to 80 ng/ml in GM. (**B**) Quantification of PTEN protein expression when BMP-2 was increased from 0–80 ng/ml after normalized to GAPDH (consider as 100%). (**C**) Typical pictures of PTEN and pAKT protein expressions as a function of time when BMP-2 was supplemented at 40 ng/ml in GM. Quantification of PTEN (**D**) and pAKT (**E**) protein expressions after normalized to GAPDH (consider as 100%). Reduced PTEN protein expression was found when GW9662 was added alone (**F**) or combined with BMP-2 (**G**). Reduced PTEN protein expression was also found when bpV (HOpic) was added alone (**H**) or combined with BMP-2 (**I**). (*: vs 0 ng/ml BMP-2, p<0.05; &: vs 0 hour, p<0.05).

Western blot analysis suggested that PTEN protein expression level was BMP-2 dose dependent (Figure 5A). It was found that the highest PTEN protein expression level was achieved when BMP-2 was between 40–60 ng/ml concentrations when PASMCs were cultured in GM supplemented with BMP-2 for 8 hours (Figure 5A&B).

Next, PASMCs were cultured in GM supplemented with 40 ng/ml BMP-2 for a serial time (Figure 5C&D). PTEN protein expression level significantly increased between 4–8 hours in presence of BMP-2 (Figure 5C&D). Reduction of pAKT was found after addition of BMP-2 (Figure 5C&E). AKT protein expression was unchanged in presence of BMP-2.

To determine whether PTEN inhibitor or PPARγ antagonist will inhibit or block the effect of BMP-2 on PTEN production, PASMCs were pre-treated with 2.5 uM bpV (HOpic) (PTEN inhibitor) or 1 uM GW9662 (PPARγ antagonist) for 1 hour before addition of BMP-2. It was found that bpV (HOpic) and GW9662 reduced PTEN protein expression (Figure 5F-I).

### QRT-PCR and Western Blot Analysis for Smad-4 Gene and Protein Expression

To determine whether BMP-2 mediated PTEN up-regulation was mediated by Smad signalling pathway, a plasmid carrying siRNA-Smad-4 was used to inhibit Smad-4 gene expression. Lipofectamine-2000 was used as a transfection vehicle to encapsulate siRNA-Smad-4 plasmid (Lipo-siRNA-Smad-4).

**Figure 6:**
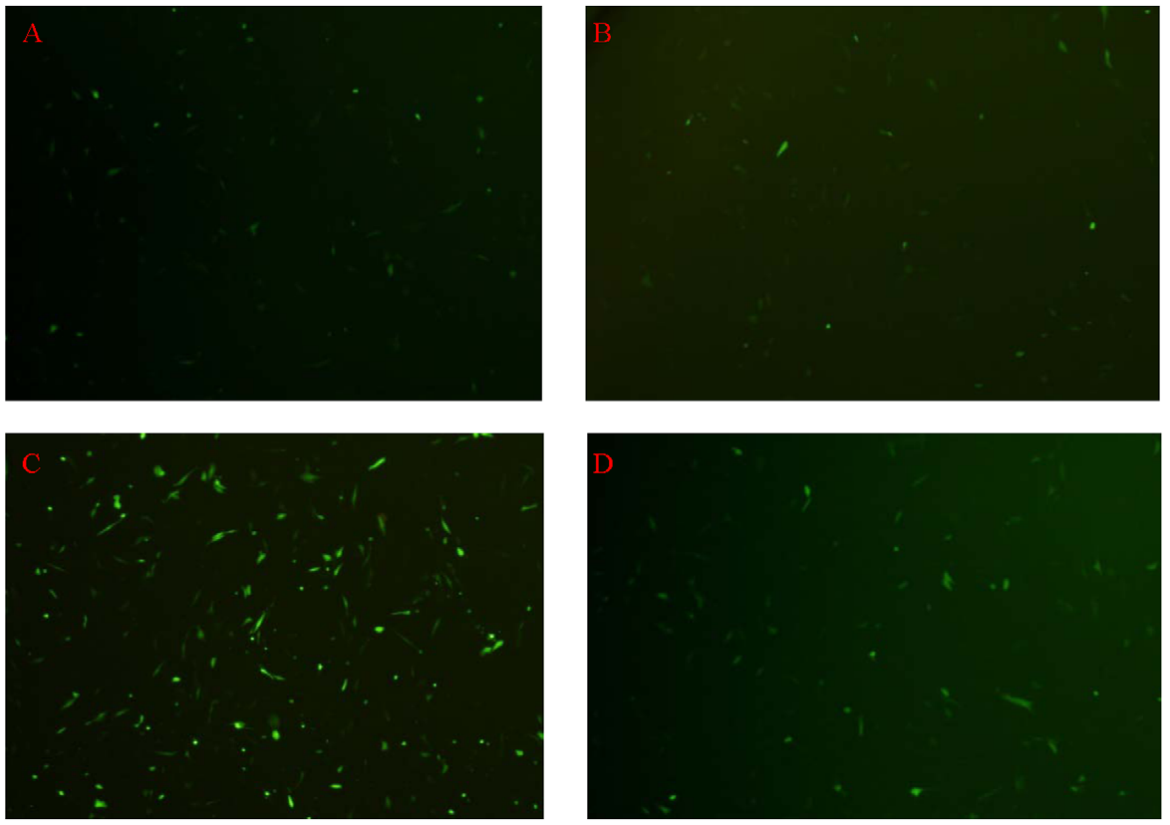
Typical pictures of EGFP expression from pEGFP lipoplexes transfected PASMC when the ratio between Lipofectamine-2000 and plasmid DNA was at (A) 1∶1, (B) 2∶1, (C) 3∶1 and (D) 4∶1, when 2 µg plasmid DNA was used per 1×10^5^ cells. (Magnification = 40×).

The transfection condition was first optimized using a plasmid carrying enhance green fluorescent protein (pEGFP). Lipo-pEGFP was added into cell culture medium for 24 hours. Then gene transfection efficiency was determined (Figure 6). It was found that the highest gene transfection and expression efficiency was achieved when 6 µl Lipofectamine-2000 was used to carry 2 µg pEGFP (V/W = 3∶1) to transfect 1×10^5^ trypsinized PASMCs. Typical EGFP gene expression pictures of PASMCs after transfection with Lipo-pEGFP were shown in Figure 6.

**Figure 7:**
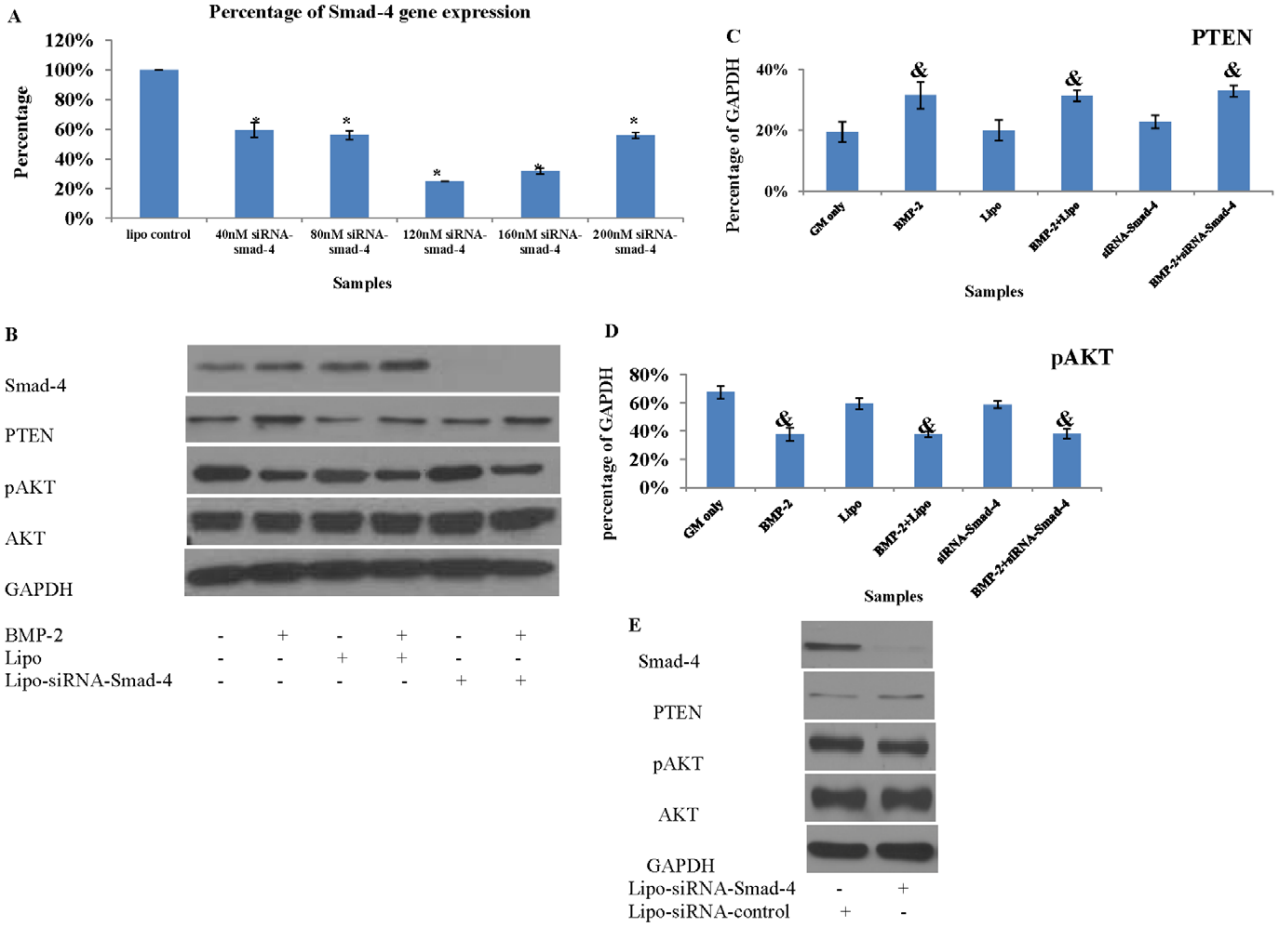
Smad-4 gene and protein expressions of Lipo-siRNA-Smad-4 transfected PASMCs. (**A**) QRT-PCR demonstrated that the lowest Smad-4 gene expression was achieved when 120 nM siRNA-Smad-4 was used to transfect 1×10^5^ PASMCs. (**B**) Abolishment of Smad-4 protein expression did not affect PTEN protein expression. Qantification of PTEN (**C**) and pAKT (**D**) protein expression after normalized to GAPDH (consider as 100%). (**E**) A non-coding siRNA was used as a negative control to determine the specificity of siRNA-Smad-4 targeting. (Lipo = lipofetamine-2000. *: vs lipo only, p<0.05; &: vs GM only, p<0.05).

Based on the optimized transfection condition, PASMCs were transfected with lipo-siRNA-Smad-4. QRT-PCR for Smad-4 gene expression demonstrated that Smad-4 gene expression was successfully reduced to 25% of non-transfected cells when 6 µl Lipofectamine-2000 was used to carry 120 nM siRNA-Smad-4 (Figure 7A). This was confirmed by Western blot analysis showing that Smad-4 protein expression level was significantly reduced after siRNA-Smad-4 gene transfection (Figure 7B).

It was found that BMP-2 induced up-regulation of PTEN protein expression was not abolished even when Smad-4 protein expression was significantly reduced (Figure 7B-C). BMP-2 still significantly reduced pAKT protein level when Smad-4 was significantly reduced. (Figure 7D). This suggests that the up-regulated PTEN protein expression by BMP-2 was not mediated by Smad-4 signalling pathway.

### The Effects of bpV(HOpic) and GW9662 on Proliferation of PASMC

**Figure 8:**
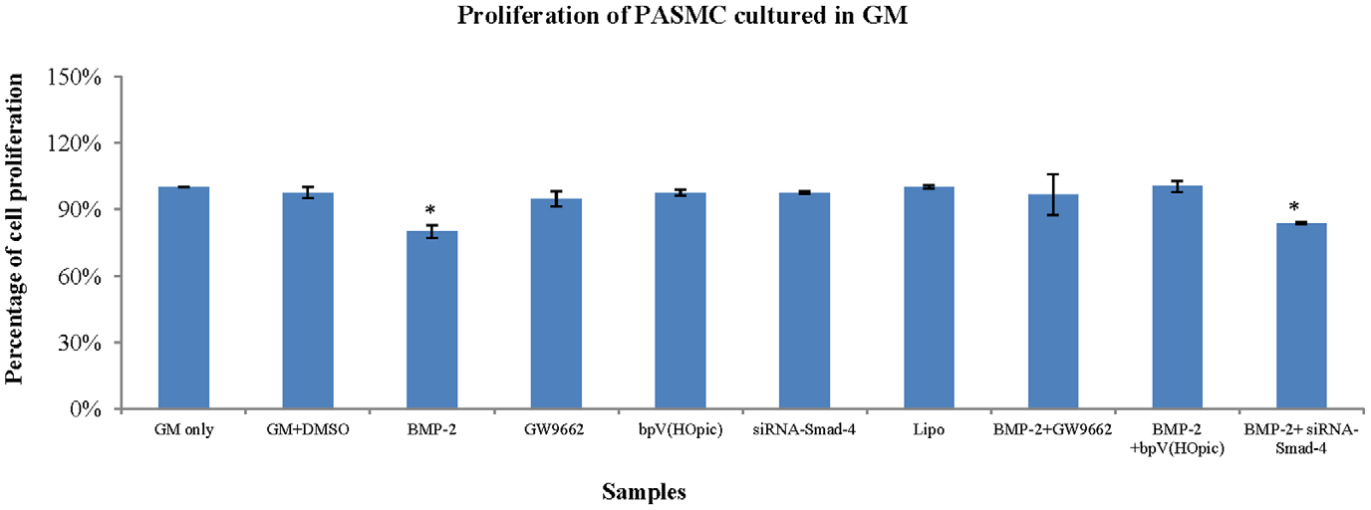
Growth profile of PASMCs cultured in GM supplemented with BMP-2, GW9662 (PPARγ antagonist) or bpV(HOpic) (PTEN inhibitor). PPARγ antagonist and PTEN inhibitor were added into respective cell culture medium 1 hour before adding BMP-2 (40 ng/ml). (The number of PASMCs after cultured in GM only was considered as 100%). (*: vs GM only) (Lipo = Lipofetamine −2000).

To determine whether PTEN inhibitor could reverse the anti-proliferative effect of BMP-2 on PASMC, bpV (HOpic) was included in cell culture medium for 72 hours under hypoxia (Figure 8). It was found that addition of bpV(HOpic) recovered PASMCs proliferation rate, suggesting it abolished the anti-proliferative effect of BMP-2 on PASMCs. Combining the results of western blot, these suggest that BMP-2 up-regulated PTEN expression, which could be reversed by PTEN inhibitor.

GW9662 was also included in cell culture medium to investigate whether the up-regulated PTEN was through PPARγ signalling pathway (Figure 8). It was found that GW9662 reversed the anti-proliferative effect of BMP-2 on PASMCs. Combining with western blot results, these suggest that BMP-2 could up-regulate PTEN expression, which is through BMP-2/PPARγ signalling pathways.

### BMP-2 Increased Caspase -3, -8, and -9 Activities

**Figure 9:**
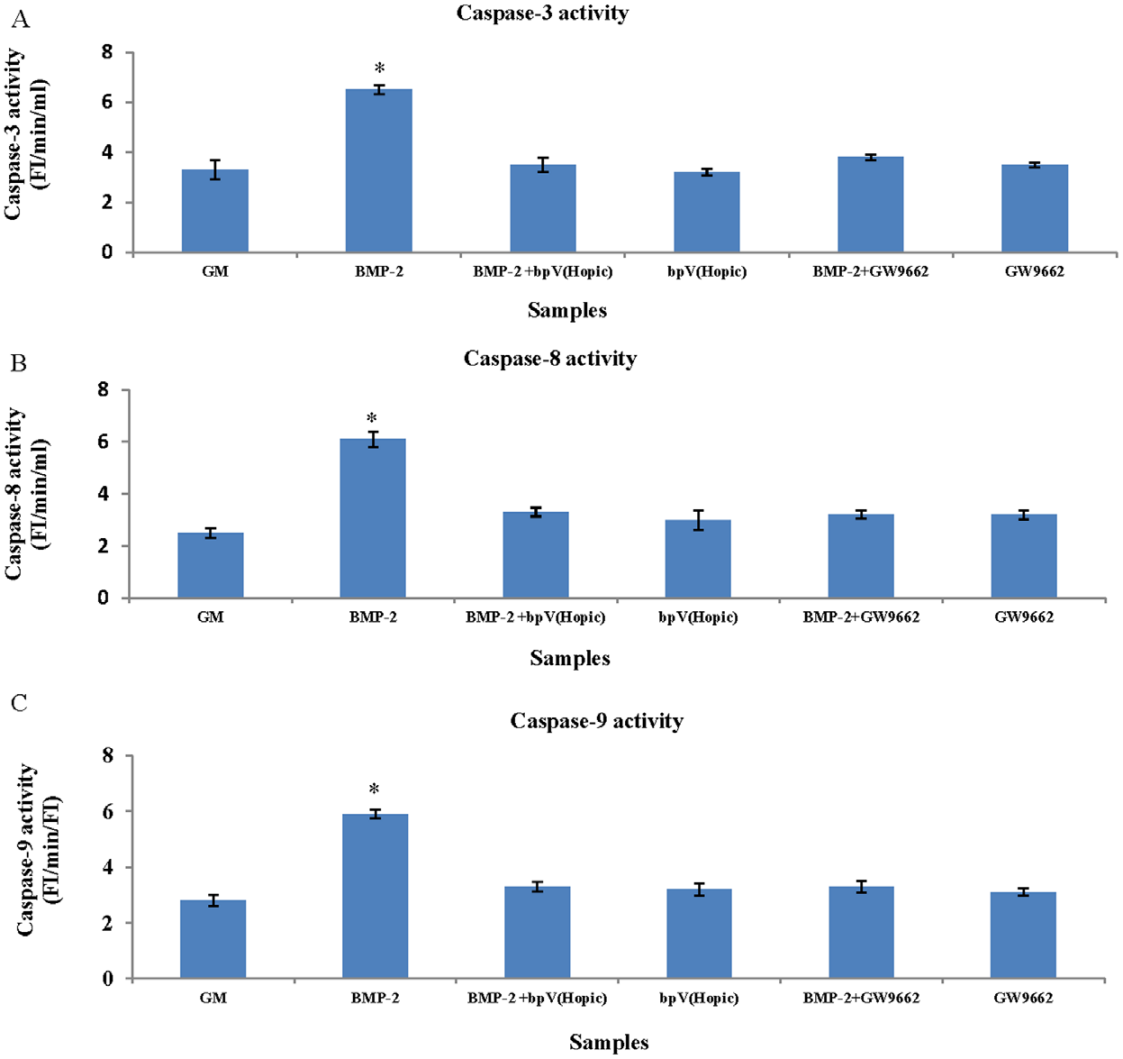
BMP-2 increased caspases 3, 8, and 9 activities. BMP-2 (40 ng/ml) significantly increased caspases 3 (**A**), 8 (**B**), and 9 (**C**) activities of PASMCs cultured in GM. However, pre-treat PASMCs with GW9662 and bpV(HOpic) reversed this effect (*: vs other samples, p<0.05).

Significantly increased caspase-3 activity was found when PASMCs were cultured in medium (6.5±0.18 FI/min/ml) supplemented with BMP-2 for 8 hours (Figure 9A). Similarly, significantly increased caspase-8 activity was also found when PASMCs were cultured medium (6.1±0.3 FI/min/ml) supplemented with BMP-2 for 8 hours (Figure 9B).

Significantly increased caspase-9 activity was found when PASMCs were cultured in medium (5.9±0.17 FI/min/ml) supplemented with BMP-2 for 8 hours (Figure 9C).

These suggested that BMP-2 increased caspase activity and may promote apoptosis of PASMC under hypoxia.

## Discussion

The present study demonstrated that BMP-2 up-regulated PTEN gene and protein expression levels of PASMCs under hypoxia. BMP-2 increased caspase activities of PASMCs under hypoxia. The increased PTEN expression was mediated through BMP-2/PPARγ signalling pathway.

The pulmonary vascular remodelling in pulmonary arterial hypertension is characterized by changes in pulmonary vascular structure [1], [2]. This is partially caused by the imbalanced PASMC proliferation and apoptosis [11]. Increased proliferation and decreased apoptosis of PASMC results in thickening of the pulmonary vasculature, which subsequently increases pulmonary vascular resistance, and pulmonary artery pressure [12].

BMP-2 has been shown to inhibit human aortic SMCs [13] and rat vascular SMCs proliferation [3]. Morrell and colleagues [14] demonstrated that BMP-2 at doses of 1–100 ng/ml inhibited [3H] thymidine incorporation in PASMCs from normotensive and secondary pulmonary hypertension patients cultured in media that contained FBS. Our results are consistent with these findings. BMP-2 of 40–60 ng/ml concentrations most efficiently inhibited PASMC proliferation under hypoxia. It significantly reduced PASMC proliferation rate under GM condition. The anti-proliferative effect of BMP-2 could be related to its role in up-regulating of PTEN and increasing caspase activity of PASMCs under hypoxia.

BMP-2 can activate Smad signalling system through BMP receptor-II (BMP-RII) [15]. To determine whether the up-regulated PTEN expression could also be mediated by Smad pathway, gene expression of Smad-4 was inhibited using siRNA-Smad-4. Smad-4 is a universal co-factor for Smad -1, -2, -3, -5, and -8. It was found that when the expression of Smad-4 was inhibited, PTEN protein expression was still up-regulated. This suggested that BMP-2 mediated up-regulation of PTEN expression was independent of Smad signalling pathway.

In the current study, BMP-2 up-regulated PTEN gene and protein expression levels. The up-regulated PTEN expression can be inhibited by PPARγ antagonist suggesting the up-regulated PTEN gene and protein expressions were mediated by PPARγ signalling. We propose here that BMP-2 mediated up-regulation of PTEN of PASMC under hypoxia was through PPARγ signalling. Hassman et al., [5] demonstrated that anti-proliferative effect of BMP-2 was BMP-RII, PPARγ, and apoE dependent. Others found that BMP-2 exposure can regulate PTEN protein levels by decreasing PTEN’s association with the degradative pathway [16]. Teresi et al., [17] demonstrated that transcriptional activation of PPARγ by lovastatin or rosiglitazone could increase PTEN expression. Our data, at least in part, is consistent with these and supports that BMP-2 through PPARγ signalling up-regulates PTEN expression that play an important role in anti-proliferation of PASMC.

Current study also suggested that BMP-2 could increase caspase activity of PASMC under hypoxia. It is known that chronic hypoxia can prolong the growth of human vascular SMC by inducing telomerase activity and telomere stabilization [18]. Zhang et al., [19] showed that BMP-2 treatment increased pulmonary vascular SMC apoptosis, by TUNEL assay, via decreasing Bcl-2 mRNA and protein expression. Lagna et al., [20] demonstrated that BMP can activate apoptotic signalling cascade via BMP receptor II. The current study is consistent with these findings. It was found that BMP-2 increased activities of caspases -3, -8 and -9. The increased caspase activity can induce the apoptosis of PASMC under hypoxia.

In summary, the study highlights that BMP-2 can increase PTEN expression under hypoxia in a dose dependent pattern. BMP-2 can increase caspase activities of PASMC under hypoxia. The increased PTEN expression may be mediated through PPARγ signalling pathway, instead of BMP/Smad signalling pathway.
